# Mode‐of‐action analysis of the effects induced by nicotine in the *in vitro* micronucleus assay

**DOI:** 10.1002/em.22314

**Published:** 2019-08-30

**Authors:** Daniel J. Smart, Fabian R. Helbling, Maëlle Verardo, Damian McHugh, Patrick Vanscheeuwijck

**Affiliations:** ^1^ PMI R&D Philip Morris Products S.A. Neuchâtel Switzerland

**Keywords:** aneugenicity, lysosomotropism, genotoxic mechanism

## Abstract

Nicotine's genotoxic potential has been extensively studied *in vitro*. While the results of mammalian cell‐based studies have inferred that it can potentially damage chromosomes, in general and with few exceptions, adverse DNA effects have been observed primarily at supraphysiological concentrations in nonregulatory assays that provide little information on its mode‐of‐action (MoA). In this study, a modern‐day regulatory genotoxicity assessment was conducted using a flow cytometry‐based *in vitro* micronucleus (MN) assay, Good Laboratory Practice study conditions, Chinese hamster ovary cells of known provenance, and acceptance/evaluation criteria from the current OECD Test Guideline 487. Nicotine concentrations up to 3.95 mM had no effect on background levels of DNA damage; however, concentrations above the point‐of‐departure range of 3.94–4.54 mM induced increases in MN and hypodiploid nuclei, indicating a possible aneugenicity hazard. Follow‐up experiments designed to elucidate nicotine's MoA revealed cellular vacuolization, accompanying distortions in microtubules, inhibition of tubulin polymerization, centromere‐positive DNA, and multinucleate cells at MN‐inducing concentrations. Vacuoles likely originated from acidic cellular compartments (e.g., lysosomes). Remarkably, genotoxicity was suppressed by chemicals that raised the luminal pH of these organelles. Other endpoints (e.g., changes in phosphorylated histones) measured in the study cast doubt on the biological relevance of this apparent genotoxicity. In addition, three major nicotine metabolites, including cotinine, had no MN effects but nornicotine induced a nicotine‐like profile. It is possible that nicotine's lysosomotropic properties drive the genotoxicity observed *in vitro*; however, the potency and mechanistic insights revealed here indicate that it is likely of minimal physiological relevance for nicotine consumers. Environ. Mol. Mutagen. 2019. © 2019 The Authors. *Environmental and Molecular Mutagenesis* published by Wiley Periodicals, Inc. on behalf of Environmental Mutagen Society.

## INTRODUCTION

Nicotine is one of the principal alkaloids found in tobacco plants, and it represents a prevalent chemical constituent of cigarettes, smoking cessation aids, inhalers, oral tobacco products, heated tobacco products, and electronic nicotine delivery systems (Federal Food, Drug, and Cosmetic Act 2009). Nicotine's genotoxic potential has been the subject of numerous investigations over the past four decades. While data generated in the bacterial reverse mutation (Ames) test consistently demonstrated that nicotine is non‐mutagenic and therefore purport a lack of direct DNA reactivity (Florin et al. 1980; Riebe et al. 1982; Doolittle et al. 1995; Yim and Hee 2001), studies carried out in mammalian cells have generally inferred that it has the potential to damage chromosomes. Historical hazard identification (ID)‐type Chinese hamster ovary (CHO) cell line‐based assays, designed to flag the effects of nicotine on sister chromatid exchange and chromosome aberrations, reported significant genotoxic effects on these endpoints (Riebe and Westphal 1983; Trivedi et al. 1990, 1993). Similarly, other *in vitro* rodent cell studies revealed an apparent causal relationship between nicotine exposure and DNA damage (Mailhes et al. 2000; Barley et al. 2004; Sudheer et al. 2007a, 2007b). The translatability of these rodent cell effects to human cell systems has also been illustrated (Kleinsasser et al. 2005; Ginzkey et al. 2013; Sobkowiak et al. 2014; Yu et al. 2016). Moreover, *in vitro* organotypic cell cultures (e.g., nasal, gingival, and respiratory cells) that partially recapitulate the cellular environment that is first exposed in human nicotine consumers have been shown to contain various forms of DNA damage following nicotine exposure (Argentin and Cicchetti 2004; Kleinsasser et al. 2005; Sassen et al. 2005; Ginzkey et al. 2009, 2010; Demirhan et al. 2011; Ginzkey et al. 2012, 2014a, 2014b). There is also some evidence to suggest that nicotine may have aneugenic properties, as revealed by data from studies that examined numerical and structural chromosome changes in exposed human and rodent cells (Racowsky et al. 1989; Zenzes and Bielecki 2004; Liu et al. 2007; Demirhan et al. 2011). In contrast, a minority of studies reports non‐genotoxic findings in mammalian cells (Bishun et al. 1972; Altmann et al. 1984; Doolittle et al. 1995), including one *in vitro* MN study conducted in human lymphocytes up to a concentration of 10 mM (European Chemical Agency (ECHA). 2017).

Although the genotoxicity of nicotine has been detected using a multiplicity of endpoints across diverse mammalian cell lines throughout this extensive body of literature, in general and with few notable exceptions (e.g., Demirhan et al. 2011), the two prevailing consistencies between these studies are (1) the supraphysiological range of concentrations (≥1 mM) required to elicit adverse DNA effects and (2) the lack of mechanistic information demonstrating how the damage was induced. It is also apparent from the paucity of recent evidence that the genotoxic potential of nicotine has been evaluated to contemporary regulatory standards only minimally (i.e., one set of studies published in the ECHA database). Regulatory genotoxicity testing is a dynamic field of toxicology, driven by internationally agreed assays that are refined periodically by authoritative bodies and expert working groups (e.g., the Organization for Economic Co‐operation and Development (OECD) and International Workshops on Genotoxicity Testing) (Kirkland et al. 2007a; OECD. 2009). Furthermore, when these assays are conducted under the Good Laboratory Practice (GLP) quality system, the data generated from them not only are mutually acceptable between countries but, moreover, hold greater weight in a regulatory context than those lacking this attribute (Klimisch et al. 1997; OECD. 1998). Various elements pertaining to the core battery of mammalian genotoxicity assays have also been improved recently, such as the choice of cell lines, exposure conditions, and generic protocols, as well as acceptance and evaluation criteria, with the ultimate outcome of enhancing the detection of biologically relevant genotoxic effects while, at the same time, minimizing the frequency of spurious results (OECD, 2016a, 2016b Test Guidelines (TG) 487 and 490). Crucially, there are some examples of chemicals (e.g., benzofuran, monuron, daminozide, 2‐mercaptobenzothiazole, and toluene) whose historical genotoxic classification was overturned when they were retested according to current consensus recommendations (Kirkland and Fowler 2010). While progress has been made in many facets of assay conduct, it should also be recognized that these assays are primarily used for hazard ID purposes, where high concentrations and appreciable levels of cytotoxicity are mandated (OECD. 2007). However, despite the questionable biological relevance associated with these aspects of this testing strategy, data can still be used to inform a chemical risk assessment by interpreting them in the context of other relevant data (i.e., the weight of evidence) and by understanding MoA and potency (Thybaud et al. 2007; White and Johnson 2016).

In view of the dearth of modern‐day genotoxicity data on nicotine, the aim of this study was to corroborate the nicotine effects previously reported in CHO cells using a state‐of‐the‐art regulatory assay, the flow cytometry‐based *in vitro* MN assay, under GLP study conditions. This approach was further bolstered by utilizing a CHO cell line with known provenance and applying current acceptance and evaluation criteria from OECD TG 487 (adopted: 29 July 2016). Based upon the results generated in this version of the *in vitro* MN assay, a series of follow‐up experiments was carried out in order to elucidate nicotine's MoA as well as to provide physiological context to these findings.

## MATERIALS AND METHODS

### Chemicals

United States Pharmacopeia/European Pharmacopeia grade nicotine (‐/L enantiomer) was obtained from Nicobrand (Coleraine, UK). All other chemicals were of the highest possible quality and obtained from Sigma‐Aldrich (Buchs, Switzerland) unless otherwise specified.

### Cell Culture

The Chinese hamster ovary‐Wolff Bloom Litton (CHO‐WBL) cell line was obtained from Merck Research Laboratories (Boston, MA) and certified as free of mycoplasma. This cell line is the same as that deposited by the International Life Sciences Institute Health and Environmental Sciences Institute Project Committee on the Relevance and Follow‐up of Positive Results in *In Vitro* Genetic Toxicity Testing (Lorge et al. 2016). Briefly, liquid nitrogen‐stored cells were thawed and then cultivated in a humidified incubator at 37°C in an atmosphere of 5% CO_2_ for at least 7 days prior to conducting an assay. Cells at sub‐confluence were passaged by trypsinization using trypsin–EDTA (Thermo Fisher Scientific, Waltham, MA), seeded at low density in cell culture flasks (75 or 175 cm^2^), and used between passages 2 and 5 in a given assay. McCoy's 5A + GlutaMAX™ medium (Thermo Fisher Scientific) supplemented with fetal bovine serum (10% v/v; Thermo Fisher Scientific), penicillin (100 U/mL), and streptomycin (100 μg/mL) was used for culture maintenance.

### *In vitro* MN Assay

#### GLP Study

On two independent test occasions, CHO‐WBL cells were seeded (4,500 cells/well) into transparent, flat‐bottom 96‐well plates (Nunc™, Thermo Fisher Scientific) and cultivated for 24 hr. Duplicate cultures were then exposed to a range of nicotine concentrations (1.97–9.86 mM) for 24 hr in the absence of S9 at 37°C and 5% CO_2_ in a humidified environment. In addition, nicotine's effects following short‐term treatment (4 hr) in the absence and presence of an S9 metabolic activation system (Aroclor‐1254 induced rat liver, Moltox, Boone, NC) and 24‐hr recovery were also investigated. After these periods (and also immediately prior to treatment; see below for more information), nuclei species, including 2n‐4n nuclei, apoptotic nuclei, hypodiploid nuclei (HDN), and MN, were harvested using the *In Vitro* MicroFlow® Kit (Litron Laboratories, Brighton, NY) in general accordance with the manufacturer's instructions. This kit is composed of reagents that fluorescently label these nuclei species and render them amenable to flow cytometric analysis (Bryce et al. 2007). Using this approach, cell cycle changes are elucidated, and in parallel, the fragmented DNA from apoptotic cells that may confound the analysis is omitted from MN enumeration (Avlasevich et al. 2006). A minor adaptation of the kit manufacturer's instructions was implemented in order to permit the measurement of an OECD TG 487‐recommended cytotoxicity index (i.e., relative population doubling [RPD]) rather than other non‐recommended indices, such as relative survival. Ten thousand 6 μm Cell Sorting Set‐up Beads (Thermo Fisher Scientific) were added to each well and used in a calculation with other flow cytometry acquisition data (nuclei events × 10,000)/bead events) to yield the absolute number of nuclei/well both immediately prior to and following treatment; these values were ultimately used to produce the RPD index of cytotoxicity applied in this study. A FACSCanto II Flow Cytometer (BD Biosciences, San Jose, CA) equipped with FACSDiva software (v8.01, BD Biosciences) was used to enumerate the frequency of each nuclei species. Concentration‐response data were analyzed statistically via (1) the Dunnett's pairwise test; (2) the Mann–Kendall trend test; (3) a comparison against the 95% upper control limit of the laboratory's historical solvent‐treated control database (SAS® Enterprise Guide® v6.1). In addition, these data were also analyzed quantitatively to produce benchmark dose (BMD) point‐of‐departure (PoD) metrics using a critical effect size (CES) of 0.5 (RIVM PROAST Web, PROAST v65.2, Netherlands).

#### Non‐GLP Experiments

For the *in vitro* MN assays carried out in the presence of chemicals that raise the luminal pH of acidic organelles, duplicate cultures were co‐exposed to the same range of nicotine concentrations and a fixed concentration of either ammonium chloride (NH_4_Cl; 5 mM), bafilomycin A1 (BafA1; 0.9 nM), or nigericin (100 nM) for 24 hr in the absence of S9 on three independent test occasions. Similarly, for experiments conducted on the major mammalian nicotine metabolites (e.g., cotinine, nornicotine (‐/L enantiomer), nicotine *N′*‐oxide, and 3′‐hydroxycotinine) (the latter three from Toronto Research Chemicals, Toronto, Canada), cells were exposed on two test occasions up to a maximum concentration of 10 mM, except for nicotine *N′*‐oxide (5 mM due to solubility limitations), prior to harvesting and enumerating the nuclei species.

### Immunostaining and Analysis

#### α‐Tubulin

To assess for potential effects on α‐tubulin‐related microtubule structure, CHO‐WBL cells were seeded at 11,000 cells/well in 8‐well chamber slides (Nunc Lab‐Tek™ II Chamber Slide™ System, Thermo Fisher Scientific) and cultivated for 24 hr. Cells were then treated with nicotine for 24 hr at 37°C and 5% CO_2_ in a humidified environment. Following exposure, cells were fixed with Cytofix/Cytoperm™ solution (BD Biosciences) for 20 min at 4°C before being washed with Perm/Wash™ buffer (BD Biosciences) and blocked with 1% w/v nonfat milk solution (NFMS) for 30 min at 37°C. Following blocking, cells were incubated with a mouse anti‐α‐tubulin monoclonal antibody (Sigma‐Aldrich; diluted 1:500 in 1% w/v NFMS) for 1 hr. Cells were then washed twice with BD Perm/Wash buffer and incubated with a goat anti‐mouse IgG (H + L) Alexa Fluor® 488 secondary antibody (Thermo Fisher Scientific; diluted 1:500 in 1% w/v NFMS) for 1 hr. Following a further washing step, the walls of the chamber slides were removed, and ProLong® Gold Antifade Mountant with DAPI (Thermo Fisher Scientific) was added before coverslips were mounted. Slides were analyzed after 24 hr on an inverted epi‐fluorescence microscope (40× objective, Eclipse Ti‐E, Nikon, Tokyo, Japan), and images were captured for manual evaluation (the absence or presence of obvious morphological changes in α‐tubulin appearance) using NIS‐Elements software (v4.51, Nikon). Unless stated otherwise, all immunostaining processing steps were performed at room temperature and in the dark.

#### Centromere

To evaluate whether the apparent fragments of DNA produced by nicotine in the *in vitro* MN assay were composed of whole chromosomes, cells were grown, treated, fixed, washed, and blocked as for α‐tubulin detection. Following blocking, cells were incubated with a human anti‐centromere protein polyclonal antibody (Antibodies, Davis, CA; diluted 1:75 in 1% w/v NFMS) for 1 hr. Following two washing steps, cells were incubated with a goat anti‐human IgG (H + L) Alexa Fluor 488 secondary antibody (Thermo Fisher Scientific; diluted 1:400 in 1% w/v NFMS) for 1 hr. Slides were then processed and analyzed manually (for the absence or presence of centromeric protein on DNA).

#### γH2AX

To investigate for effects on the clastogenicity biomarker γH2AX, cells were seeded at 130,000 cells/well in 6‐well plates and cultivated for 24 hr on three independent test occasions. Cells were then treated with nicotine or methyl methanesulfonate (MMS) for 24 hr at 37°C and 5% CO_2_ in a humidified environment. Following exposure, cells were trypsinized, and the resulting cell suspensions were then fixed with Cytofix/Cytoperm solution for 20 min at 4°C. Cells were washed free of fixative and then incubated with a rabbit anti‐phospho‐Histone H2A.X (Ser139) Alexa Fluor 647 monoclonal antibody (clone 20E3, Cell Signaling Technology, Danvers, MA; diluted 1:40 in PBS) and 7‐aminoactinomycin D (7‐AAD) DNA counterstain (BD Biosciences; diluted 1:40 in PBS) for 30 min. Cells were analyzed on the FACSCanto II Flow Cytometer, and γH2AX‐ and DNA‐associated fluorescence were collected in the Alexa Fluor 647 and 7‐AAD channels, respectively, and at least 10,000 events were acquired. Median Alexa Fluor 647 (γH2AX) fluorescence values were extracted and expressed as fold‐change in γH2AX relative to solvent‐treated controls.

#### Phospho‐serine10‐H3

To investigate for effects on the aneugenicity biomarker phospho‐serine10‐H3, cells were grown, treated with nicotine, MMS, colchicine, or paclitaxel for 4 hr, and then fixed as for γH2AX detection on three independent test occasions. Cells were washed free of fixative and incubated with a rabbit anti‐phospho‐histone H3 (Ser10) Alexa Fluor 488 polyclonal antibody (Cell Signaling Technology; diluted 1:50 in PBS) and 7‐AAD DNA counterstain (BD Biosciences; diluted 1:40 in PBS) for 30 min. Cells were analyzed as for γH2AX, except phospho‐serine^10^‐H3‐associated fluorescence was collected in the FITC channel. Data are expressed as the proportion of cells expressing phospho‐serine10‐H3 from the total cell population assessed.

### Neutral Red Uptake Assay

Cell seeding and treatment were carried out as previously described for the *in vitro* MN assay. Following treatment, cell culture medium was replaced with medium containing 50 μg/mL neutral red (NR) dye supplemented with 20 mM HEPES. After 3 hr, intracellular NR dye was extracted using a destaining solution (ethanol, water, and acetic acid, mixed in a 50:49:1 ratio), and absorbance was measured at 540 nm using a microplate reader (Safire 2 with Magellan Tracker v7.0 software, Tecan, Switzerland).

### Tubulin Polymerization Assay

The effects of nicotine on tubulin polymerization were assessed using the fluorescence‐based tubulin polymerization assay kit according to the manufacturer's instructions (Cytoskeleton, Denver, CO). Briefly, a buffer containing 2 mg/mL porcine brain tubulin, 80 mM PIPES pH 6.9, 2 mM MgCl_2_, 0.5 mM EGTA, 1 mM GTP, and 15% v/v glycerol was incubated with either 0.01–10 mM nicotine, 2.3 μM colchicine, or 3 μM paclitaxel in black, clear/flat‐bottom 96‐well plates (FluoroNunc™, Thermo Fisher Scientific) within a fluorimeter (FlexStation® 3; Molecular Devices, San Jose, CA) pre‐warmed to 37°C. Excitation and emission wavelengths of 360 and 420 nm, respectively, were used, and readings were taken every minute for a total of 60 min. Maximum velocity (*V*_max_) and maximum product values were subsequently extracted from the data using SoftMax® Pro software (v7.0 GxP, Molecular Devices).

## RESULTS

### Nicotine‐Induced Genotoxicity in the *in vitro* MN Assay

**Figure 1:**
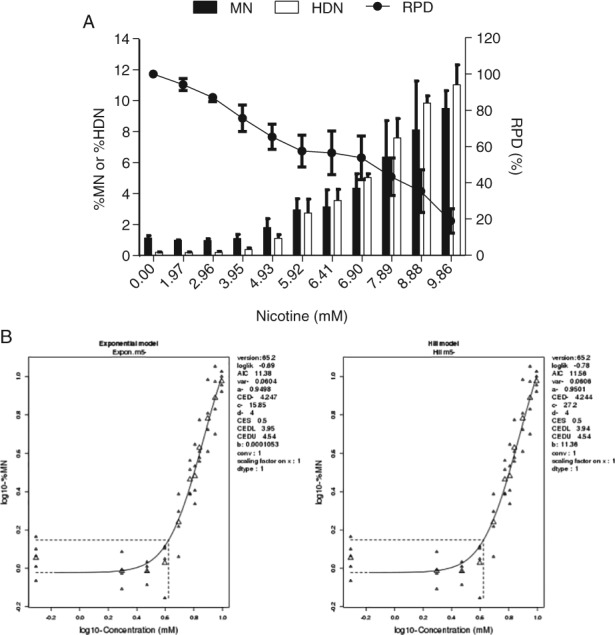
Nicotine‐induced effects in CHO‐WBL cells following 24‐hr exposure, as measured by the flow cytometry‐based *in vitro* MN assay (*n* = 2). (A) MN, HDN, and RPD endpoints. (B) BMD approach‐derived PoD for the MN endpoint.

**Figure 2:**
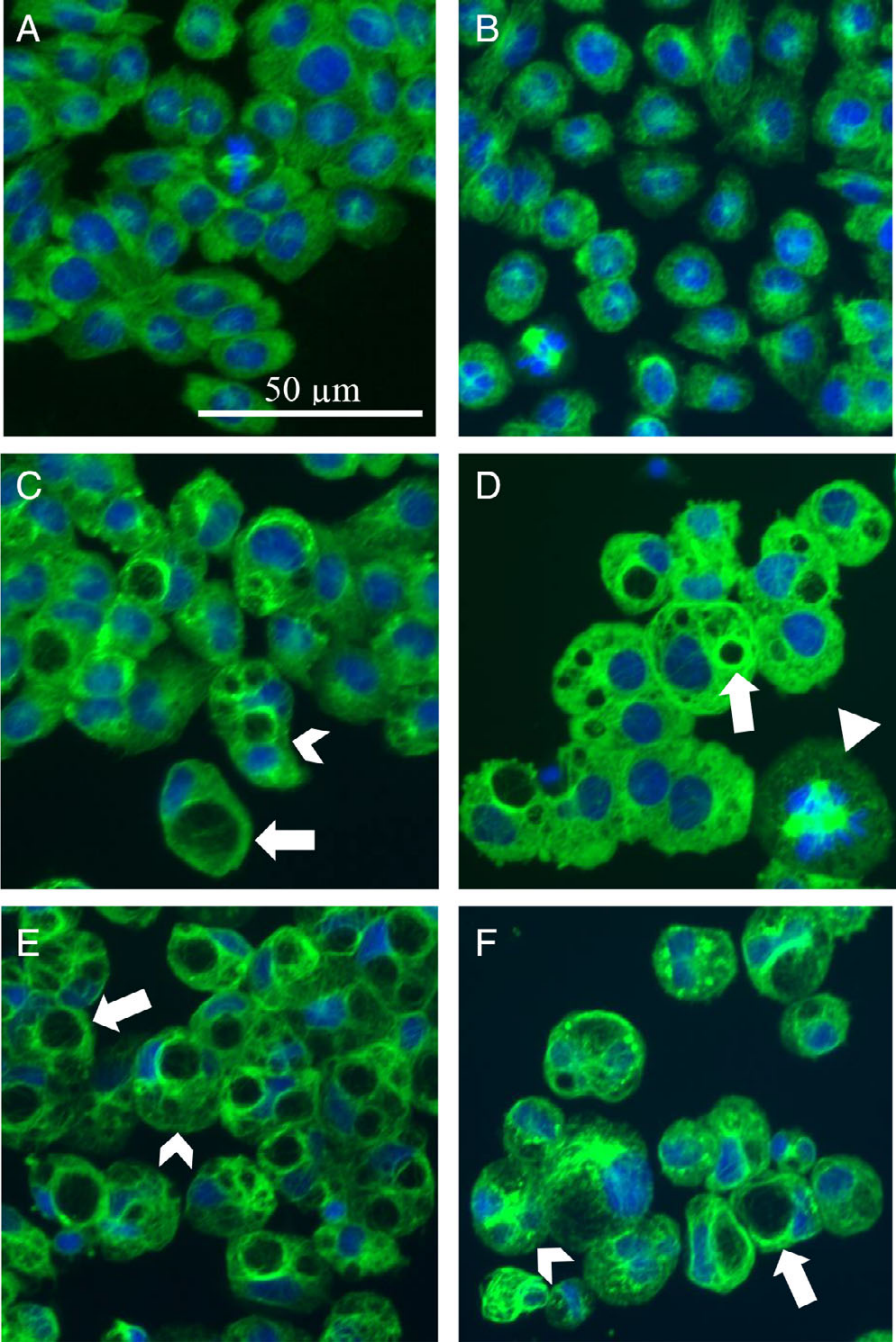
Representative images of nicotine‐induced distortions in α‐tubulin‐related microtubules (green: α‐tubulin; blue: nuclear DNA; 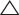 : vacuole; 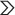 : aberrant metaphase; [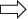]: multinuclear cell). (A) Solvent‐treated control. (B) 3.95 mM. (C) 4.93 mM. (D) 5.92 mM. (E) 6.90 mM. (F) 8.88 mM.

CHO‐WBL cells were exposed to concentrations of nicotine up to 9.86 mM for 24 hr prior to harvesting nuclei species for enumeration by flow cytometry. An apparent nonlinear response was observed; concentrations up to 3.95 mM had no discernable effect on background levels of MN; however, at concentrations ≥4.93 mM, tandem increases in MN and HDN were evident (Fig. 1A). Nicotine‐induced cytotoxicity, which was measured concurrently with genotoxicity, increased in a concentration‐dependent manner up to and beyond the assay's cytotoxic limit (i.e., RPD 40%) (Fig. 1A). In addition, the tripartite statistical approach confirmed that the MN‐related effects were statistically significant and, furthermore, the quantitative PoD analysis produced the following BMD confidence intervals (CI): 3.94–4.54 mM (%MN) (Fig. 1B) and 2.47–2.83 mM (%HDN) (Fig. S1). Nicotine was found to induce similar MN responses in the short‐term treatment experiments (±S9) (Fig. 2A,B).

### Nicotine‐Induced Vacuolization, Distortions in Microtubule Structure, and Changes in Ploidy Status

Nicotine induced negligible detectable effects on the microtubule cytoskeleton ≤3.95 mM. However, at higher concentrations, gross distortions in microtubule structure were observed (Fig. 2). At 4.93 mM, the presence of large intracellular vacuoles was conspicuous, and this presumably led to the vacuole‐shaped distortion of α‐tubulin‐related microtubules in the same cells. At concentrations ≥5.92 mM, cells were increasingly vacuolated, and a similarly distorted α‐tubulin network was also evident. In addition, while a low frequency of normal metaphase cells was detected throughout the solvent‐ and ≤3.95 mM nicotine‐treated cell cultures, there was some evidence of aberrant metaphase cells (e.g., containing tripolar spindles) detected at higher concentrations (Fig. 2). Furthermore, multinuclear cells were also evident following exposure to these higher concentrations of nicotine (Fig. 2).

### Nicotine‐Induced Reductions in the Rate and Extent of Tubulin Polymerization

**Figure 3:**
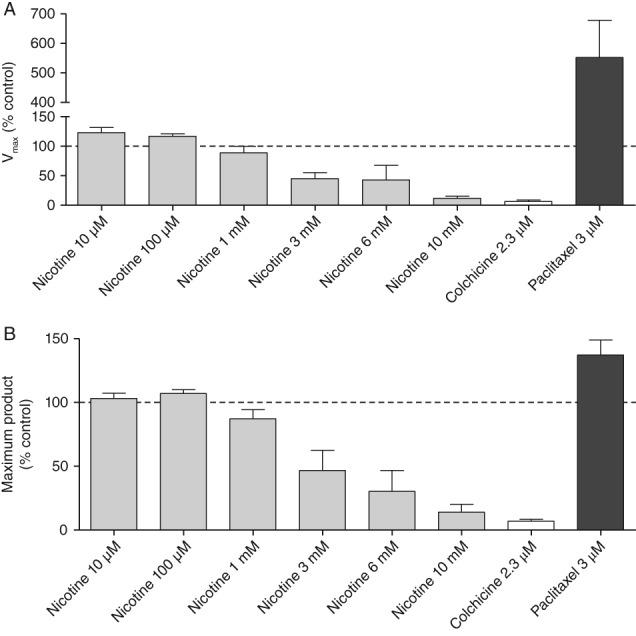
Nicotine‐induced effects on the rate and extent of tubulin polymerization in a cell‐free assay alongside the impact of colchicine and paclitaxel. (A) *V*_max_. (B) Maximum amount of tubulin produced.

A cell‐free biochemical assay was used to assess the impact of nicotine on the process of tubulin polymerization. Concentrations of nicotine from 0.01 to 1 mM had negligible effects on the rate and extent of tubulin polymerization; however, higher concentrations (3, 6, and 10 mM) produced decreases in both the *V*_max_ as well as the maximum amount of product (i.e., tubulin polymer) produced in the assay (Fig. 3A,B). The tubulin destabilizer colchicine had a similar profile in the assay, although its inhibitory effects were manifested at 2.3 μM. In contrast, the other positive control (paclitaxel, a tubulin stabilizer) at a similar concentration (3 μM) increased *V*_max_ and enhanced the extent of tubulin polymerization, as expected.

### Nicotine‐Induced Multinuclear Structures Contain Centromeric Protein

**Figure 4:**
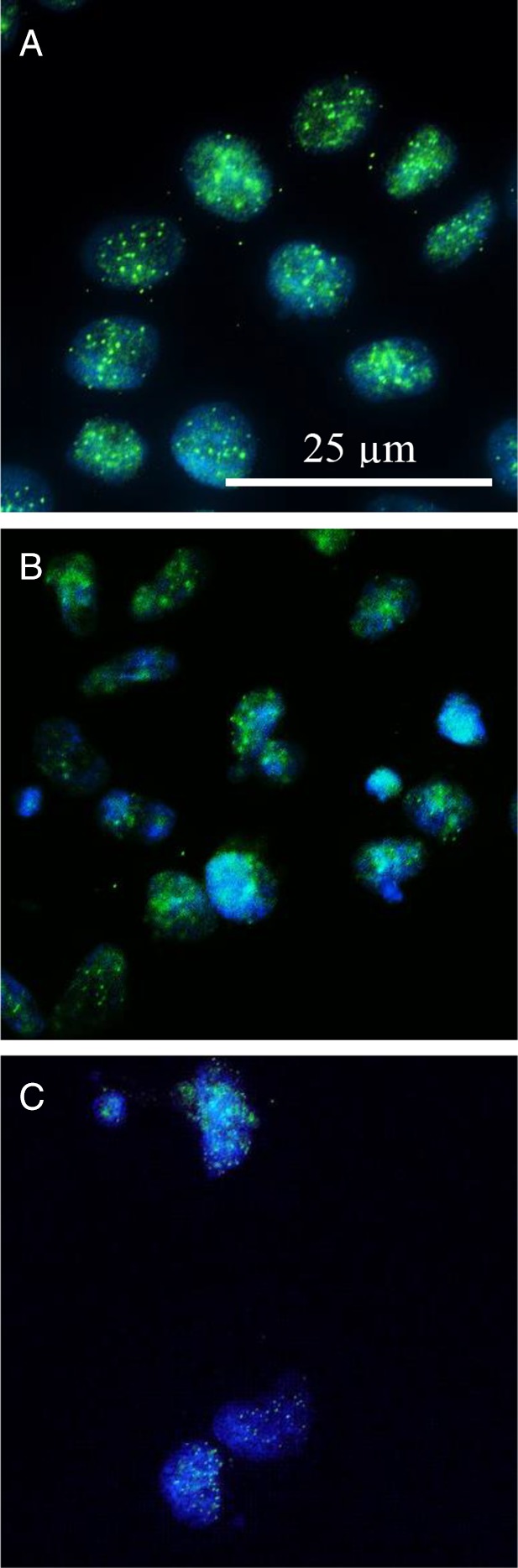
Representative images of centromere and nuclei co‐localization in nicotine‐exposed CHO‐WBL cells (green: centromere; blue: nuclear DNA). (A) Solvent‐treated controls. (B) 5.92 mM. (C) 6.41 mM.

The genomic DNA of CHO‐WBL cells exposed to genotoxic concentrations of nicotine ≥4.93 mM was probed for the presence of centromeric protein. In general, all genomic DNA identified with the DAPI nuclear stain was co‐localized with centromeric protein (Fig. 4).

### Nicotine‐Induced Vacuoles Likely Originate from Acidic Cellular Compartments

**Figure 5:**
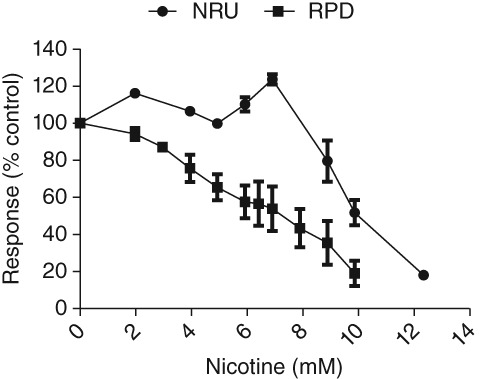
Nicotine‐induced NRU vis‐à‐vis cytotoxicity measured via RPD in the *in vitro* MN assay (*n* = 5 for the NRU endpoint; *n* = 2 for the RPD endpoint).

In order to shed light on the origin of the nicotine‐induced vacuoles, an neutral red uptake (NRU) assay, which can reveal vacuolar effects on the acidic compartments of cells, was carried out. NRU was increased relative to controls at the lower‐mid part of the concentration range (1.97–5.92 mM) before achieving a maximum relative increase of 124% (6.9 mM) and then decreasing from this peak following treatment with higher concentrations (8.88–9.86 mM) until it was abolished at the highest concentration evaluated (12.33 mM) (Fig. 5). This profile was in contrast to the concentration‐dependent increase in cytotoxicity measured in the *in vitro* MN assay using the RPD index (Fig. 5).

### Lysosomotropic Properties of Nicotine Drive the Genotoxicity Observed in the *in vitro* MN Assay

**Figure 6:**
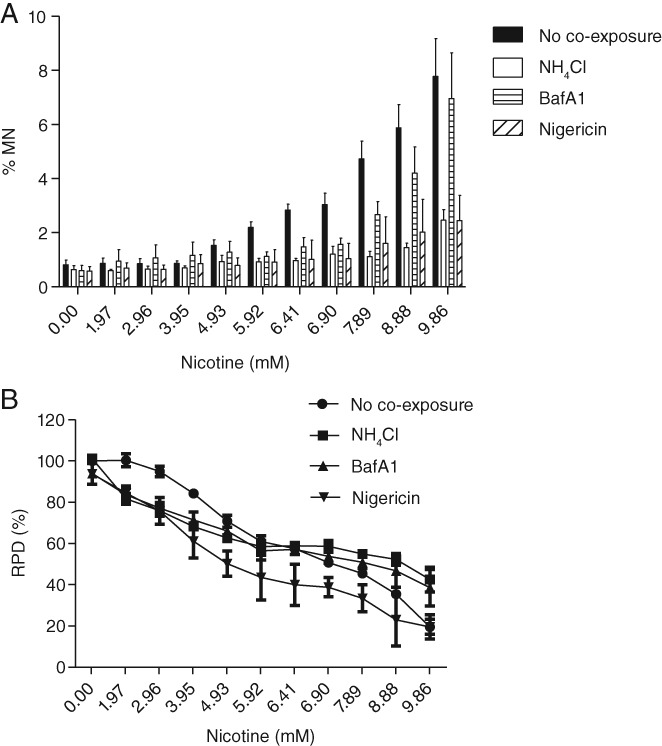
Nicotine‐induced effects in the *in vitro* MN assay in the absence and presence of lysosomal pH‐raising chemicals (*n* = 3). (A) MN endpoint. (B) RPD endpoint.

The ability of three mechanistically distinct lysosomal pH‐raising chemicals (i.e., NH_4_Cl, BafA1, and nigericin) to modulate the genotoxic potential of nicotine in the *in vitro* MN assay was investigated. While co‐exposure of the cells to nicotine and these individual chemicals did not result in a significant change in the cytotoxicity profile compared to that of nicotine alone, the MN response of the co‐exposed cell cultures was markedly attenuated, particularly at the lower effective concentrations of nicotine (4.93–6.9 mM) (Fig. 6A,B). In addition, the presence of large intracellular vacuoles was not detected microscopically in the co‐exposed cultures (data not shown). Under these conditions, NH_4_Cl was the most effective at attenuating the MN‐inducing effects of nicotine, followed by nigericin and then BafA1.

### Lack of Histone Phosphorylation and Cell Cycle Changes in Response to Nicotine‐Induced Cellular Effects

**Figure 7:**
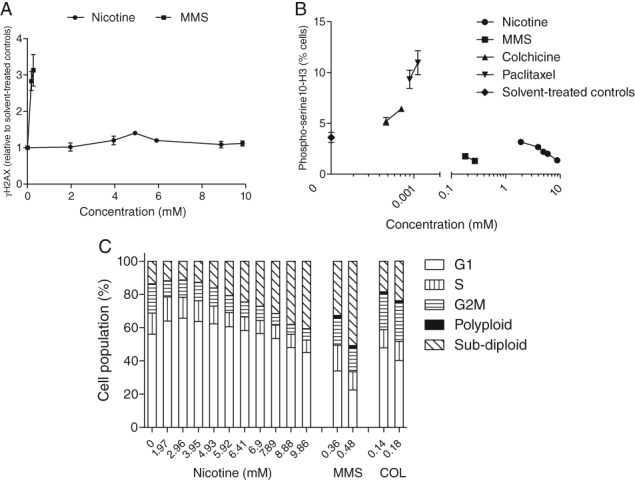
Impact of 24‐hr nicotine exposure on histone phosphorylation (*n* = 3) and cell cycle changes (*n* = 2) vis‐à‐vis the effects of the positive controls MMS, colchicine (COL), and paclitaxel. (A) γH2AX. (B) Phospho‐serine^10^‐H3. (C) Proportion of cells with G_1_, S, G_2_M, polyploid (supra‐tetraploid), and sub‐diploid (i.e., HDN and MN) DNA content.

The expression of phosphorylated histones γH2AX and phospho‐serine^10^‐H3 in cells exposed to nicotine was examined. At its maximal level, γH2AX was 1.4‐fold higher in cells exposed to 4.93 mM nicotine relative to controls, while the prototypical genotoxin MMS induced an increase up to 3.1‐fold above controls (Fig. 7A). The proportion of cells that expressed phospho‐serine^10^‐H3 decreased in a concentration‐dependent manner relative to controls following nicotine exposure (Fig. 7B). A similar effect on this biomarker was also observed in cells exposed to MMS, while, in contrast, the aneugens, colchicine, and paclitaxel increased the proportion of cells expressing phospho‐serine^10^‐H3 (Fig. 7B). Nicotine induced minimal effects on the cell cycle at all concentrations, while marked changes (e.g., G_2_M phase accumulation and endoreduplicated DNA) were observed in response to the positive controls (Fig. 7C).

### Three out of Four Major Nicotine Metabolites Were Non‐genotoxic in the *in vitro* MN Assay, while Nornicotine Induced Nicotine‐like Effects

**Figure 8:**
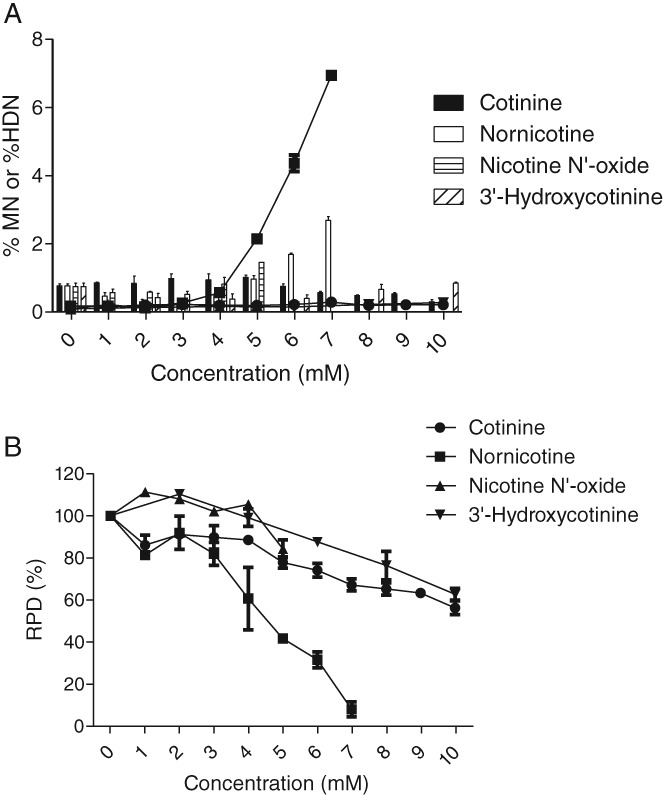
Nicotine metabolite‐induced effects in the *in vitro* MN assay following 24‐hr exposure (*n* = 2). (A) MN endpoint (bars) and HDN endpoint (lines). (B) RPD endpoint.

To provide physiological context to nicotine's genotoxic effects in the *in vitro* MN assay, four of its major mammalian *in vivo* metabolites were assessed. Up to the maximum concentrations tested, cotinine (10 mM), nicotine *N′*‐oxide (5 mM), and 3′‐hydroxycotinine (10 mM) had no detectable effects on the incidence of MN and HDN relative to controls (Fig. 8A). In contrast, nornicotine induced a nicotine‐like genotoxic response, with significant DNA effects being manifested just within the limits of cytotoxicity at concentrations ≥5 mM (Fig. 8A,B). Large intracellular vacuoles were also present at the concentrations of nornicotine that gave rise to MN effects (data not shown).

## DISCUSSION

We sought to corroborate earlier reports of nicotine‐induced genotoxicity in CHO cells by using a modern‐day approach that included (1) a state‐of‐the‐art flow cytometry‐based *in vitro* MN assay; (2) GLP study conditions; (3) a CHO‐WBL cell line of known provenance; and (4) current recommendations from OECD TG 487. While the exposure of CHO‐WBL cells to nicotine concentrations ≤3.95 mM for 24 hr had no detectable effect on the background MN frequency, levels ≥4.93 mM induced concentration‐dependent increases in MN relative to controls. Importantly, the majority of these effects were observed within the suggested cytotoxicity limits for this assay (i.e., ≥RPD 40%) and, following confirmation of their statistical significance, nicotine was classified as genotoxic in this study (similarly for the 4 hr ± S9 assessments). Thus, these data support the previous reports of nicotine‐induced genotoxicity in CHO cells (Riebe and Westphal 1983; Trivedi et al. 1990, 1993).

It was also apparent that the frequency of the HDN endpoint also measured in this version of the assay was elevated in a similar pattern to MN after 24 hr exposure. HDN represent a nuclei population that ostensibly appears “healthy” in the assay but that stains marginally less intensively with the DNA‐intercalating dye than the regular diploid population due to alterations in DNA organization (Bryce et al. 2011). In a related cell line, CHO‐K1, which has a modal chromosome number (20) similar to CHO‐WBLs (21), induction in HDN alongside MN has been shown to occur specifically in response to aneugenic chemicals (e.g., colchicine) but not clastogens; therefore, this endpoint may help to elucidate a chemical's genotoxic MoA (Bryce et al. 2010, 2011). These were the first empirical evidence of nicotine's apparent aneugenic potential at high concentrations and also guided the selection of the experiments to explore its genotoxic MoA.

The discipline of genetic toxicology has transitioned to one that now provides quantitative concentration‐response information that can ultimately be used to facilitate human risk assessment (White and Johnson 2016). The concentration‐response data generated here were analyzed quantitatively in order to establish the concentration range of nicotine at which significant genotoxic effects were manifested (i.e., the PoD). The BMD‐associated CI has previously been used as the PoD metric to support MoA determination (Wills et al. 2016). Using a conservative CES of 0.5 (i.e., a 50% increase in %MN relative to concurrent controls) (Zeller et al. 2016), the BMD CIs for nicotine‐induced %MN and %HDN were 3.94–4.54 and 2.47–2.83 mM, respectively. This lower PoD for %HDN may again support the notion that aneugenicity, with a potential nondisjunction component, is the MoA underlying nicotine's effects in the assay (Elhajouji et al. 1997). While the study has limitations (e.g., the use of the limited CHO‐WBL cell line) as well as being deployed in a hazard ID context, PoDs in this range indicate that there is a low likelihood of inducing these effects in human nicotine consumers who are exposed to concentrations several orders of magnitude lower than this (up to peaks of 0.6 and 10 μM in blood plasma and saliva, respectively) (Ginzkey et al. 2014b). Although there is a paucity of data describing the proximate concentrations of nicotine at particular sites of exposure (e.g., lung), the kinetics of its absorption and distribution are both rapid and extensive, such that any initial bolus concentration is not predicted to persist locally for long (Benowitz et al. 2009). This postulation again reaffirms low exposure levels within the human body following the utilization of nicotine products under conditions of intended use. Interestingly, nicotine's ability to damage the DNA of rodent bone marrow, blood, and liver cells has also been explored in several *in vivo* assays, for example, MN, comet, and chromosome aberrations (Bishun et al. 1972; Sen and Sharma 1991; Adler and Attia 2003; Attia 2007; Muthukumaran et al. 2008; Sudheer et al. 2008; Chattopadhyay et al. 2010; Da Silva et al. 2010; Kahl et al. 2012). The majority of these studies report genotoxic findings, however, exposure information is lacking and/or the high doses administered may have given rise to overt organ toxicity; both of which hamper the interpretation of genotoxicity data from such investigations.

In addition to quantitative concentration‐response analysis, mechanistic data on apparently genotoxic agents are integral to the comprehension of their relevance to humans. Through this information, many chemicals that would have once been categorized as “genotoxic” are now described in a more nuanced manner, and importantly, any potential concerns over their impact on human genome integrity are mitigated (Kirkland et al. 2007b). In this study, follow‐up experiments were conducted that centered on aneugenic mechanisms due to the prior clues revealed. Aneugens exert their genotoxicity via thresholded MoAs; one frequent mechanism is through the interference of the cell division apparatus (e.g., mitotic spindle), which ultimately leads to aneuploidy (Aardema et al. 1998; Parry et al. 2002). An established method of visualizing such effects is through the immunolabeling of proteins present in microtubules (e.g., α‐ and β‐tubulin) (Johnson and Parry 2008; Hernández et al. 2013). Its application here showed that nicotine concentrations ≤3.95 mM had negligible effects; however, at 4.93 mM in a proportion of cells, gross distortions in the α‐tubulin cytoskeleton were evident, and these effects were further exacerbated at higher concentrations with a profile that mirrored that of MN induction. Cells appeared vacuolated and this presumably led to the vacuole‐shaped distortions in their microtubule structure. From these experiments, there was also evidence that some cells treated with nicotine ≥4.93 mM contained aberrant metaphase nuclei (e.g., a tripolar spindle) or multinuclear structures, whereas these were not observed in controls or at lower concentrations of nicotine. Separate immunofluorescence experiments revealed that centromeric protein and the DAPI‐stained multinuclear structures were generally co‐localized; these findings usually indicate that the DNA is composed of a whole chromosome(s) rather than an acentric DNA fragment, although it should be pointed out that a quantitative assessment of these findings was not conducted which may limit the interpretation of the data (Degrassi and Tanzarella 1988; Miller and Adler 1990). Furthermore, results from the tubulin polymerization assay suggest that nicotine can interfere with tubulin formation at concentrations >1 mM. These lines of evidence again point toward an aneugenic MoA, as these molecular changes have been causally linked with aneugenic agents previously (Degrassi and Tanzarella 1988; Miller and Adler 1990; Johnson and Parry 2008; Bryce et al. 2010, 2011; Hernández et al. 2013; Stock et al. 2018). Although the nicotine‐aneugenicity relationship has been reported before (Racowsky et al. 1989; Zenzes and Bielecki 2004; Liu et al. 2007; Demirhan et al. 2011), this is the first study to identify a specific molecular change that has the biological plausibility to lead to these effects. Only oxidative stress‐based MoAs have been suggested before, and while it is also possible especially at high concentrations, this mechanism is unlikely to result in these specific manifestations of toxicity (Yildiz 2004; Wu et al. 2005; Prabhulkar and Li 2010; Ginzkey et al. 2012; Schweitzer et al. 2015).

Nicotine‐induced vacuoles in exposed cells were abundantly clear upon microscopic inspection but their origin was initially unknown. However, from nicotine's physicochemical properties and literature reports, it was possible to deduce that they likely emanated from acidic cellular compartments (e.g., lysosomes). Nicotine is a weakly basic amine and has been shown to accumulate intracellularly via its lysosomotropic properties (Barlow and Hamilton 1962; Thyberg and Nilsson 1982; Thyberg et al. 1983; Jin and Roomans 1997; Gao et al. 2016). Lysosomotropism is a phenomenon whereby basic chemicals in a non‐ionized state penetrate cellular membranes but then become trapped inside the H^+^‐rich environments of acidic compartments upon protonation (Siebert et al. 2004). Our deductions were substantiated by the findings from the NRU assay, which essentially measures the storage capacity of the acidic depots of living cells. At nicotine concentrations that caused moderate levels of cytotoxicity, NRU was increased up to 124% relative to controls. These cells were highly vacuolated, and moreover, the vacuoles appeared replete with the brownish pigmentation of NR. Similar results were also observed in several other mammalian cell lines (Cudazzo et al. 2019). These effects are presumably the end result of a cascade triggered by nicotine partitioning in acidic compartments that subsequently leads to osmotic changes, organelle swelling, and/or coalescence, and finally, increased NR storage capacity (Ohkuma and Poole 1981; Olivier et al. 1995; Goldman et al. 2009; Cudazzo et al. 2019).

While, to our knowledge, these data are the first direct evidence of the relationship between lysosomotropism and microtubule distortion, others have shown that lysosomotropism‐mediated vacuolization can be diminished by modulating the pH of acidic organelles (Morissette et al. 2004; Kazmi et al. 2013). By raising the luminal pH of these compartments, chemicals are no longer readily protonated and therefore not subjected to the sequestering effects of pH partitioning. In this study, CHO‐WBL cells were co‐exposed to nicotine and a constant concentration of one of three lysosomal pH‐raising chemicals before the *in vitro* MN assay was conducted. NH_4_Cl is a weakly basic chemical that raises intracellular pH through buffering (Kazmi et al. 2013). In contrast, BafA1 and nigericin both directly target the membrane of acid compartments, with the former inhibiting vacuolar H^+^ ATPase and the latter disrupting membrane potential (Bowman et al. 1988; Steinberg et al. 2010). All three chemicals had remarkable attenuating effects on nicotine's capacity to induce MN and HDN formation in the assay, particularly at the lower effective concentrations. This strongly infers that nicotine's lysosomotropic properties are responsible for driving the effects in this assay.

Despite the convincing evidence that indicates nicotine is aneugenic in CHO cells, other data from this study tend not to support this conclusion. First, minimal effects on γH2AX and phospho‐serine^10^‐H3 histone endpoints were observed throughout the concentration range of nicotine used. These phosphorylated histones participate in the DNA damage response that is mounted in response to genotoxic agents and are increasingly used to explore genotoxic MoA (Cheung et al. 2015). γH2AX is induced following the formation of DNA double‐strand breaks and facilitates their repair (Rogakou et al. 1998). While phospho‐serine^10^‐H3 occurs in mitotic‐phase cells and is reported to increase specifically in response to microtubule‐disrupting aneugens (Hendzel et al. 1997; Bryce et al. 2014). In this study, although activation of these proteins by the positive controls occurred, γH2AX only increased to a maximal level of 1.4‐fold above controls in response to nicotine before declining at higher concentrations. Importantly, this response fell short of biological relevance according to published criteria (Smart et al. 2011; Smart and Lynch 2012; Bryce et al. 2017). Furthermore, the levels of phospho‐serine^10^‐H3 measured post‐nicotine exposure were actually decreased and not increased as would be expected in response to a tubulin‐mediated insult. Second, the absence of notable cell cycle changes, particularly at highly cytotoxic concentrations of nicotine, is generally incongruent with the induction of genuine DNA damage in mammalian cells. The genotoxic effects of chemicals have been repeatedly shown to manifest in cells not only in the form DNA damage but also as G_2_M phase arrest and endoreduplicated DNA, and this profile was not evident in response to nicotine (Bryce et al. 2007; Smart and Lynch 2012). Third and perhaps most significantly, the observation that nicotine's cytotoxicity profile was not markedly altered when MN formation was suppressed in the presence of lysosomal pH‐raising chemicals indicates that the DNA damage reported here is potentially of limited biological relevance. These findings may provide enough compelling mechanistic evidence to challenge the genotoxic conclusions on nicotine inferred from the *in vitro* MN part of this study.

Nicotine is rapidly and extensively metabolized to several metabolites (Benowitz 1988). Its plasma half‐life averages 2 hr, and among others, four major metabolites are produced before they are further metabolized prior to excretion (Benowitz 2009). To provide physiological context to the nicotine findings, cotinine and 3′‐hydroxycotinine (≤10 mM), and nicotine *N′*‐oxide (≤5 mM) were assessed and shown to have negligible impact on MN and HDN endpoints. Nornicotine, however, produced nicotine‐like effects with a similar concentration‐response profile as well as inducing similar morphological changes. As nornicotine's physicochemical properties are predicted to be comparable to nicotine's, it is reasonable to assume that these effects are mediated via its lysosomotropic properties (Crooks 1999). As the systemic clearance of nicotine is mainly through metabolism to cotinine (70%–80%) and nornicotine formation is a minor pathway (2%–3%), there is a low possibility of nornicotine‐induced MN effects occurring under normal physiological circumstances (Kyerematen et al. 1990; Nakajima et al. 1996; Yamanaka et al. 2005).

An adverse outcome pathway (AOP) is a pragmatic method to describe a sequence of causally linked events that leads to an adverse outcome (OECD. 2018). Importantly, when based on mechanistic reasoning, it may have a role in informing risk assessment and regulatory applications (Ankley et al. 2010; Kramer et al. 2011). Thus, it is potentially informative to structure the data from this study in a rudimentary AOP‐type framework. The molecular initiating event is the lysosomotropism‐mediated swelling and/or coalescence of acidic organelles, and subsequent key events are microtubule network interference and malfunctioning of the spindle apparatus during mitosis, which ultimately results in aneugenic lesions. However, the complementary information that shows the damage is (1) generally induced by nicotine concentrations ≥1 mM; (2) possibly mediated “indirectly” via microtubule interference and, therefore, presumably thresholded, or alternatively, of uncertain biological relevance due to its lack of compatibility with several mechanistic endpoints measured here; and (3) not induced by three of the major metabolites, leads to the view that these effects are likely to be of minimal physiological relevance to human nicotine consumers.

In conclusion, the findings of this modern‐day study on nicotine corroborate the few preceding CHO cell‐based studies and the many other reports of high concentration‐related genotoxicity in mammalian cells. However, we furnish a putative MoA that is not oxidative stress‐related but mediated through nicotine's lysosomotropic properties and ultimately results in DNA damage. But given the concentration requisites, the potential mechanisms at play, and the predominate detoxifying action of metabolism, the production of these effects by nicotine is probably limited to *in vitro* systems and not relevant for higher orders of biological organization.

## AUTHOR CONTRIBUTIONS

Drs. Smart and McHugh designed the study. Dr. Smart, Mr. Helbling, and Ms. Verardo generated the study data. Dr. Smart analyzed the study data, prepared the figures, and drafted the manuscript. Drs. McHugh and Vanscheeuwijck provided important intellectual input to the manuscript. All authors approved the final manuscript.

*Accepted by—*S. Smith‐Roe

## Supporting information


**Supplementary Fig. 1** BMD approach‐derived PoD for the HDN endpoint following 24‐h nicotine exposure in CHO‐WBL cells.
**Supplementary Figure 2.** Nicotine‐induced effects in CHO‐WBL cells as measured by the flow cytometry‐based *in vitro* MN assay (n = 2). A. Four‐hour exposure (‐S9). B. Four‐hour exposure (+S9).
