# Supplementary material for: Mode‐of‐action analysis of the effects induced by nicotine in the in vitro micronucleus assay
Source: Environ Mol Mutagen. 2019 Aug 30;60(9):778–91. doi: 10.1002/em.22314 (PMC6900147; doi:10.1002/em.22314)
Supplement: Supplementary file 1 — Supplementary Fig. 1 BMD approach‐derived PoD for the HDN endpoint following 24‐h nicotine exposure in CHO‐WBL cells. Supplementary Figure 2. Nicotine‐induced effects in CHO‐WBL cells as measured by the flow cytometry‐based in vitro MN assay (n = 2). A. Four‐hour exposure (‐S9). B. Four‐hour exposure (+S9). [file EM-60-778-s001.docx]

**Supplementary Data**

**Supplementary Figure 1.** BMD approach-derived PoD for the HDN endpoint following 24-hour nicotine exposure in CHO-WBL cells.

| **Supp. Figure 1.**  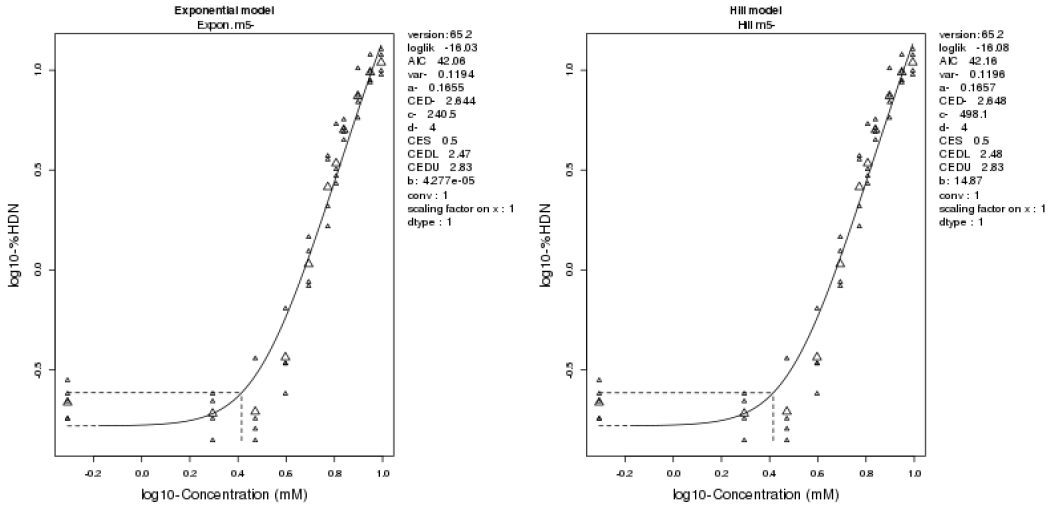 |
| --- |

**Supplementary Figure 2.** Nicotine-induced effects in CHO-WBL cells as measured by the flow cytometry-based *in vitro* MN assay (n=2). A. Four-hour exposure (-S9). B. Four-hour exposure (+S9).

| **Supp. Figure 2A.**   |
| --- |
| **Supp. Figure 2B.**   |
